# HOME AND COMMUNITY-BASED SERVICE NEEDS AMONG RURAL CARE PARTNERS OF US VETERANS: FINDINGS FROM QUALITATIVE RESEARCH

**DOI:** 10.1093/geroni/igae098.2523

**Published:** 2024-12-31

**Authors:** Elizabeth Marfeo, Elizabeth Chamberlin, Maria Venegas, Lauren Moo

**Affiliations:** Tufts University, Medford, Massachusetts, United States; Department of Veterans Affairs, Bedford, Massachusetts, United States; VA Bedford Healthcare System, Bedford, Massachusetts, United States; Department of Veterans Affairs, Bedford, Massachusetts, United States

## Abstract

The number older adults living in rural areas of the US is increasing. This demographic shift presents challenges for ensuring access to home and community-based services (HCBS) among rural older adults. We conducted a qualitative study to explore HCBS needs among family caregivers (N = 19) whose care partner is a rural older (< 65) Veteran with complex care needs via semi-structured qualitative interviews. All participants were recruited through the Veterans Health Administration’s Caregiver Support Program. An iterative content analysis was guided by Andersen’s Behavioral Model of Health Services Use. Three a priori domains for coding were initially used: psychosocial, enabling, and need factors. Key themes related to service access and use emerged including (1) significant objective and subjective health needs among both the CG and Veteran; (2) limited accessibility to health services and respite supports; (3) financial needs beyond healthcare costs; and (4) variation in attitudes, beliefs, and perceived control regarding care preferences and values from the perspective of the CG and Veteran. Most caregivers reported living in the home with the Veteran yet receiving no additional HCBS. Overall family caregivers reported taking immense pride in caring for their loved one but cited several areas of unmet needs related to accessibility and utilization of HCBS in their rural communities. These findings have important implications for identifying future pathways to increase access to HCBS, ultimately improving both rural-residing family caregiver and Veteran health and quality of life.

